# Ureteral fibroepithelial polyps in a pregnant woman: case report

**DOI:** 10.1590/S1516-31802009000400010

**Published:** 2009-12-07

**Authors:** Affonso Celso Piovesan, Fábio César Miranda Torricelli, Leonardo Lima Borges, José Luiz Chambô, José Luiz Borges de Mesquita, Miguel Srougi

**Affiliations:** 1 MD. Staff surgeon, Urology Department, Faculdade de Medicina da Universidade de São Paulo (FMUSP), São Paulo, Brazil.; 2 Resident in the Urology Department, Faculdade de Medicina da Universidade de São Paulo (FMUSP), São Paulo, Brazil.; 3 PhD. Head of the Urology Department, Faculdade de Medicina da Universidade de São Paulo (FMUSP), São Paulo, Brazil.

**Keywords:** Laparotomy, Neoplasms, Pregnant women, Ureter, Urology, Laparotomia, Neoplasias, Gestantes, Ureter, Urologia

## Abstract

**CONTEXT:**

Ureteral fibroepithelial polyps are rare benign nonepithelial tumors, and less than 200 cases have been reported in the literature. We report on a pregnant patient with ureteral fibroepithelial polyps that were successfully treated with laparotomy.

**CASE REPORT:**

A 23-year-old pregnant woman presented with a three-month history of intermittent lumbar pain of low intensity. Abdominal ultrasonography showed that she was 13 weeks pregnant and found severe left-side ureterohydronephrosis and a heterogeneous solid mass measuring 11 x 8 x 7 centimeters in the middle portion of the ureteral topography. The investigation was complemented with magnetic resonance imaging, which confirmed the previous findings. Nephroureterectomy was performed without complications. The specimen revealed three solid tumors in the ureter, of which the largest was around eight centimeters in length. The anatomopathological report confirmed that they were fibroepithelial tumors without malignant components.

## RESUMO

**CONTEXTO:**

Pólipos fibroepiteliais de ureter são tumores não-epiteliais benignos raros, e menos de 200 casos foram relatados na literatura. Nós reportamos o caso de uma mulher grávida com pólipos fibroelitelial ureteral que foram tratados com sucesso por laparotomia.

**RELATO DE CASO:**

Mulher de 23 anos de idade, grávida, apresentava história de dor lombar intermitente de baixa intensidade há três meses. Ultra-sonografia abdominal revelou gravidez de 13 semanas, ureterohidronefrose grave à esquerda e massa sólida heterogênea medindo 11 x 8 x 7 cm na porção média da topografia ureteral. A investigação foi complementada com ressonância magnética, que confirmou os achados. Foi realizada nefroureterectomia sem complicações. A peça revelou três tumores sólidos em ureter, o maior apresentando aproximadamente oito centímetros longitudinalmente. O relatório anatomopatológico confirmou tumor fibroepitelial sem componentes de malignidade.

## INTRODUCTION

Primary ureteral neoplasms are rare and account for only 1% of all upper genitourinary tract neoplasms. Benign ureteral lesions are even rarer, representing approximately 20% of all ureteral tumors, and are either epithelial or nonepithelial lesions. While papillomas or low-grade transitional cell tumors represent tumors of epithelial origin, nonepithelial neoplasms are of mesodermal origin and include benign fibromas, leiomyomas, neurofibromas, lymphangiomas, hemangiomas and fibroepithelial polyps.^1^

Ureteral fibroepithelial polyps are rare benign nonepithelial tumors that are composed of stroma derived from the mesoderm and covered by a layer of normal transitional epithelial cells. They have been documented in approximately 180 cases in published reports.^2^^,^^3^ The management of these polyps is typically dependent on the degree of obstruction, presence of any existing urinary tract infection and intraoperative suspicion of potential malignancy. It is important to distinguish fibroepithelial polyps from upper urinary tract carcinoma, because the management and prognosis may be significantly different. Today, ureteral fibroepithelial polyps can be treated by means of both open exploration and resection or minimally invasive approaches.

We report on a pregnant woman with ureteral fibroepithelial polyps that were successfully treated by means of laparotomy. We present a review of the literature on the clinical findings, diagnosis and treatment of this uncommon pathological condition.

## CASE REPORT

A pregnant 23-year-old Caucasian patient presented with a three-month history of intermittent left lumbar pain of low intensity. At the same time, she complained of a painless mass. The patient had no history of hematuria, dysuria, frequency (polacyuria), discharge of calculi or fever. Physical examination revealed a mobile, well-defined solid mass in the left lower quadrant. Urine culture and urine cytological tests were negative. Biochemical data were within normal limits.

Abdominal ultrasonography showed that the patient was 13 weeks pregnant and found severe left-side ureterohydronephrosis and a heterogeneous solid mass measuring 11 x 8 x 7 centimeters situated in the middle portion of the ureteral topography. The bladder, uterus, left uterine tube and left ovary appeared to be free of the disease. The investigation was complemented with magnetic resonance imaging, which confirmed the previous findings. No further investigation was performed because the patient was pregnant.

Open exploration was considered to be the best approach. Ureteroscopy and/or laparoscopy could have been attempted, preceding laparotomy, but this might have delayed the treatment and/or increased the operative time. We believed that a simple and faster approach would be better for the health of the fetus and mother. An extensive mass was identified in the middle portion of the left ureter, associated with severe ureterohydronephrosis. Nephroureterectomy was performed without complications. The specimen revealed three solid tumors in the ureter, of which the largest was around eight centimeters in length. The anatomopathological report confirmed that they were ureteral fibroepithelial polyps without malignant components.

The patient’s pregnancy evolved well and term birth occurred at 38 weeks of gestation without problems for either the mother or the baby. Both left the hospital fine and, after one year of follow-up, no complications were found.

## DISCUSSION

Ureteral fibroepithelial polyps are mesodermal lesions consisting of hyperplastic fibroconnective tissue with a vascular stroma and a urothelial lining. They are considered to be the most common benign tumors of the ureter but are nonetheless rare lesions. These polyps are thought to be either congenital slow-growing lesions or lesions that develop as a result of chronic urothelial irritants, such as infection, inflammation or obstruction. Less than 200 cases have currently been reported in the literature. Ureteral polyps are more common in men than women (ratio of 3:2). Most of them are solitary lesions and found in people under 50 years old. The typical length is not more than five centimeters. Hematuria is the most common presenting symptom, although other urinary symptoms may be found.^4^

Although the radiological appearance of fibroepithelial polyps is highly variable, they are frequently diagnosed exclusively by means of intravenous urography and/or retrograde ureterography. Polyps are usually viewed as long, slender and generally smooth filling defects, frequently found in the proximal ureter and sometimes associated with hydroureteronephrosis.^1^ However, the differential diagnoses between fibroepithelial polyps and malignant ureteral tumors, radiolucent calculi and blood clots remain indistinct through conventional radiographic methods. Negative findings from urinary cytological tests are helpful for ruling out transitional cell carcinoma. Sonographic methods have proved helpful for ruling out calculi and allowing soft tissue to be viewed, although images of non-dilated ureters are often suboptimal. Computed tomography can be used to differentiate fibroepithelial polyps from other radiolucent filling defects of the collecting system. Modern endoscopic techniques allow a minimally invasive approach to the proximal ureter and renal pelvis,^1^ and may provide a diagnosis. Biopsy-proven histological confirmation should be obtained for all patients before definitive therapy is started.

The treatment of choice is local resection. However, it is not uncommon for this to be replaced by nephroureterectomy. Most nephroureterectomy procedures are performed because of an uncertain preoperative diagnosis with the fear of malignant disease. Furthermore, nephrectomy is necessary when severe obstruction has destroyed the kidney completely.

Open exploration and resection was the procedure that was recommended for a long time. However, with advances in endoscopy, new options have become a reality. Lam et al.^2^ reported on a series of percutaneous, anterograde excisions of fibroepithelial polyps. Five patients underwent either percutaneous or ureteroscopic treatment of a renal pelvic or ureteral fibroepithelial polyp by means of electroresection or holmium:yttrium-aluminum-garnet laser resection. The decision regarding whether to use a percutaneous or a ureteroscopic approach was primarily based on the size and location of the fibroepithelial polyp. There was no recurrence over a mean follow-up of 19.6 months. More recently, Kijvikai et al.^5^ reported on their experience and technique for laparoscopic management of a large ureteral fibroepithelial polyp in a 42-year-old female.

## CONCLUSION

Ureteral fibroepithelial polyps are rare benign neoplasms and the literature shows that they can be treated by means of several approaches.
